# Cascading failures in coupled networks: The critical role of node-coupling strength across networks

**DOI:** 10.1038/srep35352

**Published:** 2016-10-17

**Authors:** Run-Ran Liu, Ming Li, Chun-Xiao Jia

**Affiliations:** 1 Alibaba Research Center for Complexity Sciences, Hangzhou Normal University, Hangzhou, 311121, People’s Republic of China; 2 School of Engineering Science, University of Science and Technology of China, Hefei, 230026, People’s Republic of China

## Abstract

The robustness of coupled networks against node failure has been of interest in the past several years, while most of the researches have considered a very strong node-coupling method, i.e., once a node fails, its dependency partner in the other network will fail immediately. However, this scenario cannot cover all the dependency situations in real world, and in most cases, some nodes cannot go so far as to fail due to theirs self-sustaining ability in case of the failures of their dependency partners. In this paper, we use the percolation framework to study the robustness of interdependent networks with weak node-coupling strength across networks analytically and numerically, where the node-coupling strength is controlled by an introduced parameter α. If a node fails, each link of its dependency partner will be removed with a probability 1*−*α. By tuning the fraction of initial preserved nodes *p*, we find a rich phase diagram in the plane *p−*α, with a crossover point at which a first-order percolation transition changes to a second-order percolation transition.

The infrastructures in modern life can be characterized by complex networks^1^,^2^,^3^,^4^,^5^,^6^, such as water, electricity and communication systems, the successful operations of which are ensured by the coupling between these networks. For instance, the operation of a power network requires water network for cooling power plant, transport network to supply fuel, and communication network for control, and these networks in turn require power networks to supply electricity. In order to explore the robustness of these interacting networks, the interdependent networks or multilayered networks^7^,^8^ have been studied by means of percolation^9^, which demonstrated that the interdependency exhibited a first-order percolation transition when suffering attack and makes the coupled networks more vulnerable than a single network.

The importance of node-coupling method for the network robustness has been widely recognized. For instance, reducing the fraction of coupled nodes between networks leads to a change from a discontinuous to continuous percolation transition, and thus a more robust system^10^. For multiple coupled networks, the system will be even more vulnerable^11^,^12^,^13^,^14^. Further studies have also explored the robustness of interdependent networks under different coupling methods or network structures, such as inter-similarity^15^,^16^, multiple support-dependency relations^17^, assortativity^18^,^19^,^20^, clustering^21^,^22^, degree distribution^23^,^24^, and spatially embedded networks^25^,^26^,^27^,^28^. All these works demonstrate the fragility of the coupled networks in the presence of interdependency under different situations.

However, these theoretical findings somehow conflict with the real observation of the stable coupled system. In order to understand this puzzle, some research have explored the coupling structure of real networks, which reveal that interdependent networks sharing cores of “high quality” edges^29^ as well as interconnections between network hubs^30^ can prevent catastrophic failures and make the system of networks stable. In this paper, we attribute the cause of this puzzle to a very strong node coupling across networks in the previous models and try to give a possible explanation of the stability for real-world coupled networks. In the previous studies of interdependent networks, when one node fails, it will cause its dependent node in the other network to fail at once. However, this assumption may not always true in some real situations. Although some nodes cannot run very well if their dependency nodes fail, they can hold and survive by their self-sustaining abilities. For instance, in a trading network, some companies may shrink their business by reducing the number of their trade partners if their dependent investors go bankrupt. Although this situation is very common in real-world coupled systems, there is still lack of study of this mechanism on the robustness of interdependent networks. Therefore, developing a method to analyze cases where weak node-coupling exist in the interdependent networks can help to understand the robustness of coupled complex systems in the real world as well as for designing robust infrastructures. In this paper, we will propose a model to study the robustness of interdependent networks with weak node-coupling strength between networks. By using the percolation theory^31^,^32^,^33^,^34^, we analytically calculate the discontinuous and continuous percolation transition points of our proposed model to validate the simulation results.

## Results

### Model

For simplicity and without loss of generality we analyze the percolation process in a system of two fully interdependent networks *A* and *B* with the same number of nodes *N*, whose degree distributions follow 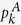 and 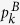, respectively. Here, the full interdependence means that all the nodes in network *A* have a mutual dependence partner in network *B*. Assuming that *a*_*i*_ from network *A* fails, each connectivity link of its dependency partner *b*_*i*_ in network *B* will be broken with a probability 1−*α*, where the introduced parameter *α* controls the impacts of the failure of its dependency partner. Similarly, if a node in network *B* fails, the connections of its dependency partner in network *A* will be also cut off as the same way. When 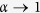, the failures cannot spread across the networks. When 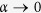, our model will reduce to the original model of interdependent networks proposed in ref. 9. Therefore, we can also define the link-removal probability 1−*α* as the node-coupling strength of two interdependent nodes.

Following the mutual percolation model described in Buldyrev *et al.*^9^, we destroy a fraction 1−*p* of randomly selected nodes in network *A*. As a result, the failures of nodes or their connectivity links may cause the other nodes to disconnect from the largest cluster of network *A*. In the next stage, each connection of a node with a failed dependency partner will fail with a probability 1−*α*. Consequently, some nodes may disconnect from the largest cluster as a result of the destruction of links in network *B*. The iteration of this process, which alternates between the two networks, leads to a cascade of failures. The cascade ends until no further splitting and node removal can occur. In our study, the sizes of giant components *S*^*A*^ and *S*^*B*^ for the final networks *A* and *B* are considered as the key quantities as the previous works^9^.

### General formalism

Here we solve this model by considering the final state after the cascades as the method of generating functions^35^,^36^. Let *R*^*A*^ be the probability that a randomly chosen link in network *A* leads to the giant component. Similarly, *R*^*B*^ is the probability that a randomly chosen link in network *B* leads to the giant component. Here, we use 
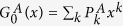
 and 
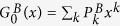
 to denote the generating functions of the degree distributions of networks *A* and *B*, respectively. Similarly, 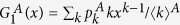 and 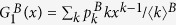 are the corresponding generating functions of the underlying branching processes of networks *A* and *B*, respectively. Then, in the steady state, *R*^*A*^ satisfies


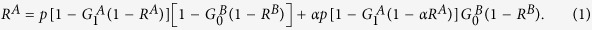


In the first term on the right-hand side, 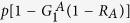 denotes the probability that a randomly chosen link starting from a randomly chosen node leads to the giant component of network *A*, and 
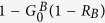
 is the probability that the dependency partner of this chosen node is still functional. In the last term, 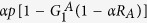 denotes the probability that a randomly chosen link starting from a randomly chosen node in network *A* leads the giant component of network *A*, and 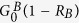 is the probability that the dependency partner of this chosen node fails. For the later case, each link of this chosen node is preserved with a probability *α*, and thus there is a coefficient *α* in this term.

Similarly, *R*^*B*^ can be written as


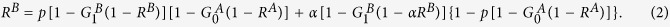


By using *R*^*A*^ and *R*^*B*^, we can easily get the probability that a randomly chosen node belongs to the giant component of the final network *A* or *B* respectively, i.e., the size of the giant component of the final networks *A* or *B*:


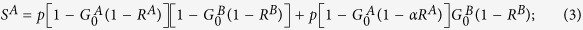



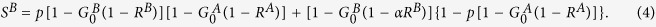


### The percolation transition points

Since the sizes of giant components *S*^*A*^ and *S*^*B*^ depend on the auxiliary parameters *R*^*A*^ and *R*^*B*^ directly, we discuss the phase transition of the system by using the parameters *R*^*A*^ and *R*^*B*^. When *α* = 1, the failure caused by the initial node removal cannot spread to network *B*, and the percolation on network *A* will reduce to the standard site percolation, which is continuous. While *α* = 0, our model is equivalent to the original model of interdependent networks, and the percolation transition is discontinuous. Therefore, we can predict that the key parameter *α* plays an important role for the percolation transition types, and the percolation transition can change from a discontinuous one to a continuous one at a crossover point *α*_*c*_ between 0 and 1. In the following, we try to locate the position of the crossover point *α*_*c*_ as well as the percolation transition points.

**Figure 1:**
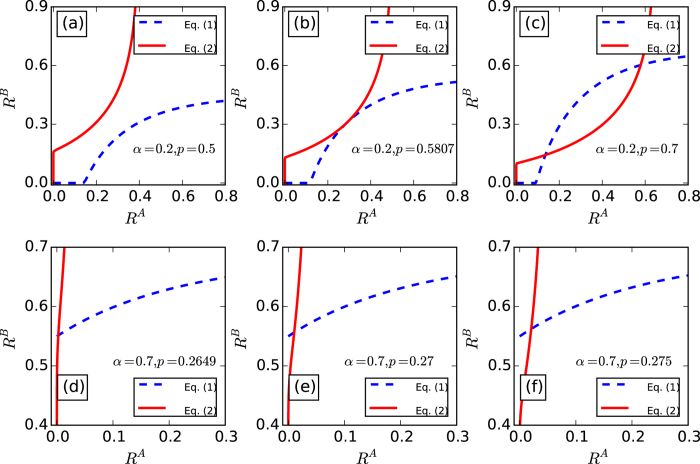
Graphical solutions of *R*^*A*^ and *R*^*B*^ for coupled random networks with 〈*k*〉^*A*^ = 〈*k*〉^*B*^ = 4. (**a–c**), *α* = 0.2 < *α*_*c*_, *p*_*c*_ ≈ 0.5807 with nonzero *R*^*A*^ and *R*^*B*^. (**d–f**), *α* = 0.7 *α*_*c*_, *p*_*c*_ ≈ 0.2649.

The solution of eqs (1) and (2) can be graphically presented on a *R*^*A*^, *R*^*B*^ plane. Here we take two coupled random networks with the same average degree as an example, the degree distribution of which follows a Poissonian distribution 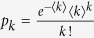
37. Figure 1 shows graphically solutions of *R*^*A*^ and *R*^*B*^ for random networks with 〈*k*〉 = 4. We notice that there is a trivial solution at the point (*R*^*A*^ = 0, *R*^*B*^ = 0), which means that the two networks *A* and *B* are completely fragmented. For *α* = 0.2, there is a tangent point of the two curves for eqs (1) and (2), the condition for which is 
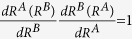
. When the fraction of preserved nodes *p* go through the tangent point, *R*^*A*^(*R*^*B*^) jumps from zero to a finite size, which corresponds to the case of first-order percolation transition. While for *α* = 0.7, we can observe that the tangent point is in absent and there is only one solution. By reducing *p*, we observe that the solution decreases continually to 0, which corresponds to a situation of second-order percolation transition. We thus distinguish types of a percolation transition as well as the first-order phase transition point 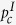 by checking the presence of the tangent point in the solution plane.

For the continuous percolation transition, we keep *R*^*B*^ constant in eq. (1), and check the behaviours of the order parameter *R*^*A*^. At the second-order phase transition point 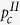, we have


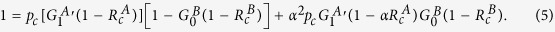


As 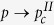, the critical value of *R*^*A*^, labeled as 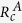, approaches to zero and the critical value of *R*^*B*^, labeled as 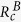, reaches it minimum 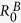. And thus we can get the continuous percolation transition point


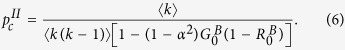


When 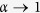, this agrees with the results in ref. 38. The typical value of 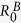 can be obtained by letting *R*^*A*^ = 0 in eq. (2), which satisfies


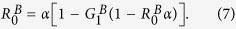


In this paper, we focus our research on the coupled random networks and scale-free networks. The random network follows a Poissonian distribution, and the scale-free network follows a distribution *P*(*k*)˜*k*^−*λ*^(*k*_*min*_ ≤ *k* ≤ *k*_*max*_), where *k*_*min*_ and *k*_*max*_ are the lower and upper bounds of the degree, respectively, and *λ* is the power law exponent. By plugging the degree distributions into the generating functions, we can explicitly get the second-order phase transition points 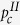 by eq. (6) and the graphical solutions for the first-order phase transition point 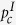. By letting 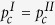, we can find the boundary between the first- and second-order phase transitions, i.e., the crossover-point value *α*_*c*_ at which there is a change from first-order to second-order percolation transition.

### Simulation results

**Figure 2:**
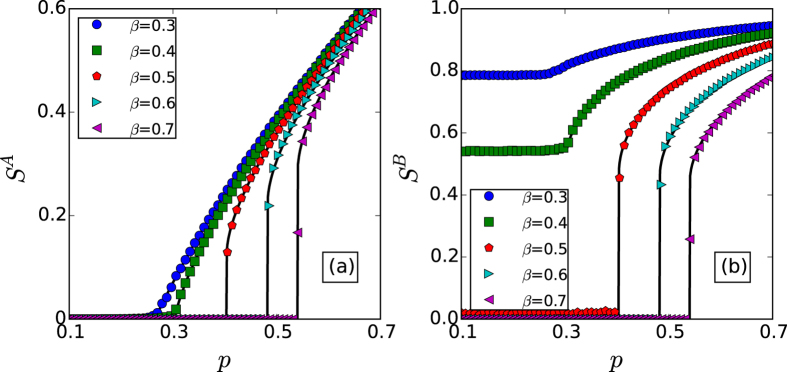
The sizes of the giant components *S*^*A*^ and *S*^*B*^ *vs. p* for coupled random networks with 〈*k*〉^*A*^ = 〈*k*〉^*B*^ = 4, respectively. The solid lines show the theoretical predictions, and the symbols represent simulation results from 20 time realizations on networks with 10^5^ nodes.

**Figure 3:**
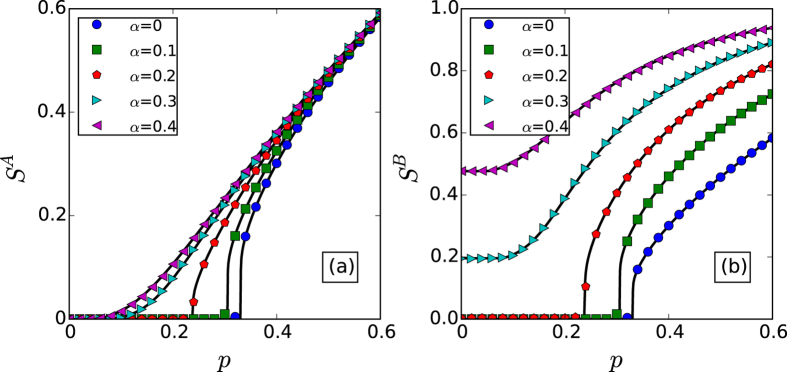
The sizes of the giant components *S*^*A*^ and *S*^*B*^ *vs. p* for scale-free networks with *k*_*min*_ = 4, *k*_*max*_ = 316 and λ = 2.7, respectively. The solid lines show the theoretical predictions, and the symbols represent simulation results from 20 time realizations on networks with 10^5^ nodes.

The varying of giant component sizes *S*^*A*^ and *S*^*B*^ in dependence on the fraction of initial preserved nodes *p* for coupled random networks are shown in Fig. 2 by both simulation and theory, from which we can find that the simulation results agree with the theory well. Moreover, we can find that there is a sharp transition of *S*^*A*^ or *S*^*B*^ from a nonzero value to zero for a small value of *α*, while for a larger value of *α*, the transition of *S*^*A*^ or *S*^*B*^ becomes continuous, which illustrates the existence of a crossover point of first-order and second-order percolation transitions as our theory predicted. Fig. 3 gives the percolation properties for coupled scale-free networks under different node-coupling strength. We can find the similar results as coupled random networks, but different percolation transition points.

**Figure 4:**
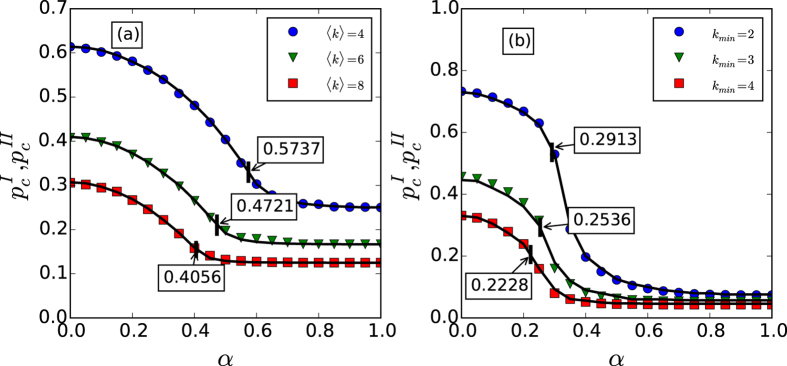
The percolation transition points *P*_*c*_, including *P*_*c*_^*Ι*^ and *P*_*c*_^*II*^, for different values of *α*. Panel (a) shows the results for coupled random networks with different average degree. The critical points *α*_*c*_ are about 0.5737, 0.4721 and 0.4056 for 〈*k*〉 = 4, 〈*k*〉 = 6 and 〈*k*〉 = 8 respectively. Panel (b) shows the results for coupled scale-free networks with different lower bounds *k*_*min*_ and the same upper bound *k*_*max*_ = 316. The critical points *α*_*c*_ are about 0.2913, 0.2536 and 0.2228 for *k*_*min*_ = 2, *k*_*min*_ = 3 and *k*_*min*_ = 4 respectively. In both panels, the symbols represent simulation results from 20 time realizations on networks with 10^5^ nodes, and error bars are comparable to the size of the symbols. The critical points *α*_*c*_ are the theoretical results by letting 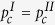, and the solid lines represent the theoretical predictions.

Figure 4 gives the percolation transition points *p*_*c*_ versus *α* for both 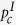 and 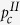. The percolation transition point can be numerically identified by the maximum fluctuation for the size of the giant component, as they are expected to be large for both first- and second-order percolation transitions^18^. From Fig. 4, one can find that the simulation and theoretical results are consistent well, as well as the existence of a crossover point *α*_*c*_, which illustrates reducing the coupling strength between interdependent nodes leads the change from a first-order percolation transition to a second-order percolation transition. Meanwhile, we can also find that a large value of *α* always leads to a small value of *p*_*c*_ for both random networks and scale-free networks, which means a weak node-coupling strength between networks make a system composed with coupled networks robust. When the parameter *α* enters the second-order percolation transition area, we find that the percolation transition point 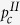 is always small and becomes insensitive to *α*, which means that when *α* exceeds the crossover point *α*_*c*_, the coupled system is always robust.

## Conclusions

In summary, we have studied the cascading failures in coupled networks with different node-coupling strength for both random networks and scale-free networks. In the previous models of coupled networks, each pair of interdependent nodes with a complete coupling strength, i.e., one of them fails, the other one will fail immediately^9^,^10^,^15^,^16^,^39^,^40^,^41^. However in our model, all nodes in one network are coupled with their counterparts in the other network, and the node-coupling strength is controlled by the link-preserved probability of a node when its interdependent partner fail. Our model is also very different from that of partially coupled networks in refs 10,40, where only a fraction of nodes that depend on the ones in the other network and the left nodes are the autonomous ones.

Our studies show rich phase transition phenomena when the model parameter *α* changes. The coupled system is robust and is characterized by a second-order transition if *α* > *α*_*c*_, while if *α* < *α*_*c*_, the coupled system is fragile and the cascading failures suggest a first-order transition. We have used the generating function method to solve our model and get the first-order and second-order percolation transition points analytically, which agree with simulation results very well. Our results prove that reducing the coupling strength between interdependent nodes can also lead to a change from a first-order to second-order percolation transition for interdependent networks even with all coupled nodes. At the same time, we have find that the second-order percolation transition point is always small and insensitive to the model parameter *α*, which means that when *α* exceeds the critical point *α*_*c*_, the coupled system is always robust. Therefore, the crossover point *α*_*c*_ that separating first-order and second-order phase transition areas has another implication, which is that *α*_*c*_ is also a split point of fragile and robust areas. This result is very different with that in ref. 15 and may be of significance for the system design by tuning the node-couple strength across networks. Furthermore, our results for interdependent networks with a weak node-coupling strength also favour the observation that real coupled systems are stable. Since the weak node coupling may be widespread in real world, our study represents an important step for characterizing the robustness properties of real coupled networks and also provide a possible explanation for the stability of real networks.

## Additional Information

**How to cite this article**: Liu, R.-R. *et al.* Cascading failures in coupled networks: The critical role of node-coupling strength across networks. *Sci. Rep.* **6**, 35352; doi: 10.1038/srep35352 (2016).
